# Ecology shapes the genomic and biosynthetic diversification of Streptomyces bacteria from insectivorous bats

**DOI:** 10.1099/mgen.0.001238

**Published:** 2024-04-16

**Authors:** Manuela Montoya-Giraldo, Kathryn R. Piper, Odion O. Ikhimiukor, Cooper J. Park, Nicole A. Caimi, Debbie C. Buecher, Ernest W. Valdez, Diana E. Northup, Cheryl P. Andam

**Affiliations:** 1 Department of Biological Sciences, University at Albany, State University of New York, Albany, New York, USA; 2 Department of Molecular, Cellular and Biomedical Sciences, University of New Hampshire, Durham, New Hampshire, USA; 3 Department of Biology, University of New Mexico, Albuquerque, New Mexico, USA; 4 Buecher Biological Consulting, Tucson, Arizona, USA; 5 U.S. Geological Survey, Fort Collins Science Center, Fort Collins, Colorado, USA

**Keywords:** bats, biosynthetic gene clusters, genome, hybrid BGC, *Streptomyces*

## Abstract

*Streptomyces* are prolific producers of secondary metabolites from which many clinically useful compounds have been derived. They inhabit diverse habitats but have rarely been reported in vertebrates. Here, we aim to determine to what extent the ecological source (bat host species and cave sites) influence the genomic and biosynthetic diversity of *Streptomyces* bacteria. We analysed draft genomes of 132 *Streptomyces* isolates sampled from 11 species of insectivorous bats from six cave sites in Arizona and New Mexico, USA. We delineated 55 species based on the genome-wide average nucleotide identity and core genome phylogenetic tree. *Streptomyces* isolates that colonize the same bat species or inhabit the same site exhibit greater overall genomic similarity than they do with *Streptomyces* from other bat species or sites. However, when considering biosynthetic gene clusters (BGCs) alone, BGC distribution is not structured by the ecological or geographical source of the *Streptomyces* that carry them. Each genome carried between 19–65 BGCs (median=42.5) and varied even among members of the same *Streptomyces* species. Nine major classes of BGCs were detected in ten of the 11 bat species and in all sites: terpene, non-ribosomal peptide synthetase, polyketide synthase, siderophore, RiPP-like, butyrolactone, lanthipeptide, ectoine, melanin. Finally, *Streptomyces* genomes carry multiple hybrid BGCs consisting of signature domains from two to seven distinct BGC classes. Taken together, our results bring critical insights to understanding *Streptomyces*-bat ecology and BGC diversity that may contribute to bat health and in augmenting current efforts in natural product discovery, especially from underexplored or overlooked environments.

**Impact Statement**

Members of the bacterial genus *Streptomyces* are the world’s most important natural source of drugs or drug precursors with broad industrial, agricultural and pharmaceutical applications, from treatment of infectious diseases and other medical disorders to biocatalysis and bioconversion systems. Our analyses of *Streptomyces* genomes derived from insectivorous bats reveal a highly diverse group characterized by the presence of multiple species harbouring a myriad of biosynthetic gene clusters that have the potential to produce new specialized metabolites. Our findings shed light in our understanding of the ecology of microbe-derived natural products, the ever-growing species diversity of *Streptomyces*, and the adaptation of microbial communities in caves and bats.

## Data Summary

The dataset supporting the conclusions of this article is included within the article and its supplementary files. Genome sequence data of the 73 previously published *Streptomyces* genomes can be found in the NCBI Sequence Read Archive (SRA) under BioProject accession number PRJNA673820. The genome sequence data of the 59 newly sequenced genomes can be found in BioProject accession number PRJNA1010360. BioSample accession numbers, associated metadata, and genomic features for each genome are listed in Table S1 (available in the online version of this article).

## Introduction

An important source of drugs or drug precursors with broad pharmaceutical and industrial applications, including the most effective antibiotics, are natural products produced by members of the genus *Streptomyces* [1]. These are Gram-positive bacteria that exhibit a complex fungal-like life cycle consisting of vegetative hyphae, aerial hyphae, and spores [2,3]. Many bioactive natural products derived from *Streptomyces* have been developed into compounds with antibacterial, antiviral, cytotoxic and antitumor, immunosuppressive, antifungal, and cellulolytic activities [4]. These clinically important compounds are derived from the secondary metabolites produced by *Streptomyces* [1,2]. Secondary metabolites are encoded in biosynthetic gene clusters (BGCs), which are physically linked genes that function together in peptide assembly, regulation, resistance, and synthesis of secondary metabolites [5].

The species diversity of *Streptomyces* is staggering, with a total of 729 validly published species (https://lpsn.dsmz.de/genus/streptomyces; as of August 2023). Many species of *Streptomyces* are found as free-living inhabitants of the soil and are known to play a critical role in carbon recycling and biodegradation [6,7]. Some are found in extreme environments [8] and in pristine and nutrient-limited ecosystems [9]. Environmental conditions in nutrient-limited ecosystems such as caves shape microbial communities toward antibiotic production to reduce competition [10]. Some *Streptomyces* also form a symbiotic association with invertebrates such as beewolf digger wasps [11], ants [12,14], and beetles [15,16], whereby the insects use the chemical compounds produced by *Streptomyces* for defence [17], e.g. protection of their eggs and larvae against opportunistic pathogens [11]. Vertebrates have not been as frequently sampled as invertebrates for *Streptomyces*, but bats have been reported to harbour *Streptomyces* on their skin surface [18,20]. The role of *Streptomyces* in bats may be significant, yet limited data are available to ascertain whether they form a symbiotic relationship with bats.

Previous studies highlight two notable features of bat-associated *Streptomyces*. First, bats represent a hitherto overlooked reservoir of vast *Streptomyces* diversity. Previously, we identified 15 novel species using a five-locus sequence analysis from a collection of 632 *Streptomyces* isolates sampled from different bat species in Arizona and New Mexico, USA [18]. In a subsequent analysis of 73 randomly selected isolates from this same culture collection, we delineated 41 different species based on genomic similarity [21]. Second, bat-associated *Streptomyces* have been reported to inhibit the growth of *Pseudogymnoascus destructans* [18], a fungus that causes the skin infection called white-nose syndrome in North American bats [22,24]. *P. destructans* disturbs the bat’s hibernation period during winter, resulting in dehydration, starvation, and often death [22,24]. It has devastated and even led to the collapse of many bat populations [25,26]. Hence, bats and caves may potentially serve as a fertile reservoir for novel *Streptomyces* species with potent antimicrobial bioactive compounds against white-nose syndrome [18]. Evidence that the skin microbiota of bats underlies a possible mechanism for resistance against white-nose syndrome has been reported [27].

Nonetheless, the evolutionary and ecological drivers that shape the genetic and species diversity of bat-associated antibiotic-producing *Streptomyces* remain unclear. Here, we sought to determine to what extent the bat host species and cave sites influence the genomic and biosynthetic diversity of *Streptomyces* bacteria. We analysed high quality draft genome sequences of 132 *Streptomyces* isolates sampled from 11 insectivorous bat species from six sites consisting of multiple caves across Arizona and New Mexico, USA. Altogether, our results show that bat-associated *Streptomyces* are remarkably diverse and their genomic and biosynthetic diversity are greatly influenced by their ecological and geographical sources.

## Methods

### Sampling and isolation of *Streptomyces* isolates

A total of 132 isolates of *Streptomyces* bacteria were analysed in the current study, of which 73 isolates were previously sequenced by our group and are publicly available in the National Centre for Biotechnology Information (NCBI) Sequence Read Archive (SRA) [21] (Table S1). Isolates were randomly chosen from a culture collection of *Streptomyces* from healthy bats (i.e. free of white-nose syndrome) sampled in 2013–2016, of which a subset (*n*=632) has been described elsewhere [18]. Details on bat collection protocols, sampling permits, and bacterial isolation procedures have been described previously and were approved by the institutions and licensing committees in reference [18]. We followed the approved protocols under the following collection permits: 2014 Arizona and New Mexico Game and Fish Department Scientific Collecting Permit (SP670210, SCI#3423, and SCI#3350), National Park Service Scientific Collecting Permit (CAVE-2014-SCI-0012, ELMA-2013-SCI-0005, ELMA-2014-SCI-0001, and PARA-2012-SCI-0003), USGS Fort Collins Science Centre Standard Operating Procedure (SOP) 2013–01, and an Institutional Animal Care and Use Committee (IACUC) permit from the University of New Mexico (protocol #12–100835-MCC) and from the National Park Service (protocol #IMR-ELMA.PARA-Northup-Bat-2013.A2).

Briefly, bats were caught using mist nets or were hand-plucked from cave walls. Bats were swabbed from caves post-hibernation or from netting on the surface of caves near their drinking sources. Four actinobacterium selective media were used to isolate *Streptomyces* (Actinomycete isolation agar [Difco, Sparks, Maryland, USA], gellan gum agar, humic acid-vitamin agar, and glucose yeast extract agar), which were supplemented with cycloheximide, nalidixic acid, trimethoprim and a vitamin solution. Agar plates were inoculated immediately after swabbing the bats and kept at 4°C during transport to the laboratory, after which they were transferred to 20°C incubator for 2–4 days. Initial *Streptomyces* identification was done by comparing sequence variation in the 16S rRNA locus carried out using Sanger sequencing [18].

### Genomic DNA extraction and whole genome sequencing

*Streptomyces* DNA was extracted using the Quick-DNA Fungal/Bacterial Miniprep Kit (ZYMO Research) following the manufacturer’s protocol. DNA concentration and quality were measured using a Nanodrop spectrophotometer and Qubit four fluorometer. Genome sequencing was carried out using the NextSeq2000 platform at the SeqCenter (Pittsburgh, Pennsylvania, USA) in 2022. Sample libraries were prepared using the Illumina DNA Preparation kit and IDT 10 bp UDI indices following the manufacturer’s instructions. Sequencing generated paired-end reads (2×151 bp) on multiplexed libraries. Demultiplexing, quality control, and adapter trimming were carried out using the Illumina bcl-convert v3.9.3.

### Genome assembly, quality check, annotation and species delineation

Raw Illumina paired-end reads were assembled into contigs using the Shovill pipeline v.1.1.0 (https://github.com/tseemann/shovill), which uses the SPAdes assembly algorithm v.3.15.2 [28]. All assembled genomes had ≤600 contigs (Fig. S1). Genome quality was assessed using QUAST v.5.0.2 [29] and CheckM v.1.1.6 [30]. We calculated the genome completeness (mean=99.91 %; range=99.24–100 %) and genome contamination (mean=1.26 %; range=0.00–2.85 %), which were all within the genome quality standards recommended by CheckM (Table S1). To delineate species boundaries, we calculated the genome-wide average nucleotide identity (ANI) for every possible pair of genomes using fastANI v.1.32 [31]. ANI refers to the mean nucleotide identity of all orthologous genes shared between a pair of genomes [31]. We used the ≥95 % ANI threshold to confirm that genomes are of the same species [31]. The draft genomes were annotated using Prokka v.1.14.6 [32].

### Pan-genome analysis and phylogenetic tree reconstruction

Using Panaroo v.1.2.7 [33], we identified all the genes that were present in the entire dataset (referred to as the pan-genome [34]). Core genes were defined as those present in ≥95 % of the genomes, while accessory genes were those present in <95 % of the genomes. Gene sequences were aligned using MAFFT v.7.505 [35]. Sequence alignments of the core genes were concatenated to generate the core genome alignment. Single nucleotide polymorphisms (SNPs) were extracted from the core genome alignment using snp-sites v.2.5.1 [36]. The core SNP alignment was used as input for building a maximum likelihood phylogenetic tree using RAxML v.8.2.12 [37]. We used the general time reversible model for nucleotide substitution [38] with the GAMMA model of rate heterogeneity. Phylogenetic trees were visualized and annotated using the Interactive Tree of Life [39].

### Identification of BGCs

BGCs encoding secondary metabolites were predicted and annotated using the standalone version of antiSMASH v.6.0.1 [40] with --genefinding-tool none and a relaxed stringency level (--hmmdetection-strictness relaxed) as recommended for common detection of bacterial BGCs. AntiSMASH identifies BGCs using a signature profile Hidden Markov Model based on multiple sequence alignments of experimentally characterized signature proteins or protein domains [40]. AntiSMASH first identifies the sequences for the primary core enzymes of a specific BGC and then identifies the secondary core gene neighbourhood upstream and downstream of the primary core gene. Thus, antiSMASH can identify a BGC even in contig edges or overlapping contigs based on the location of the core genes of a BGC. AntiSMASH defines hybrid clusters either as a single BGC which produces a hybrid compound from the combination of two or more protein scaffold types or two separate BGCs that are in close proximity [40]. Here, we do not distinguish between these two types. Individual BGC components of hybrid BGCs were also tallied. For example, the hybrid terpene-t1PKS was counted as one terpene and one t1PKS (type one polyketide synthase). We also distinguished the variants within each BGC class, e.g. NRPS (non-ribosomal peptide synthetase) consisted of NRPS only, NRPS-like, and thioamide-NRP. We also manually checked the contigs on which the BGCs were detected by antiSMASH to ensure that we are not double counting the BGCs. We used RStudio [41] to create and visualize all plots, and polished using Linearity Curve (https://www.linearity.io).

### Statistical analysis

The coefficient of determination (R^2^) was calculated using the ggpubr package [42] in R [43]. Statistical significance was measured using the non-parametric Mann-Whitney U test (also known as Wilcoxon rank sum test) and ANOVA implemented in R [43]. We used a *p*-value threshold ≤0.05 to consider significance of our results.

## Results

### Bat-associated *Streptomyces* are phylogenetically diverse

**Fig. 1.**
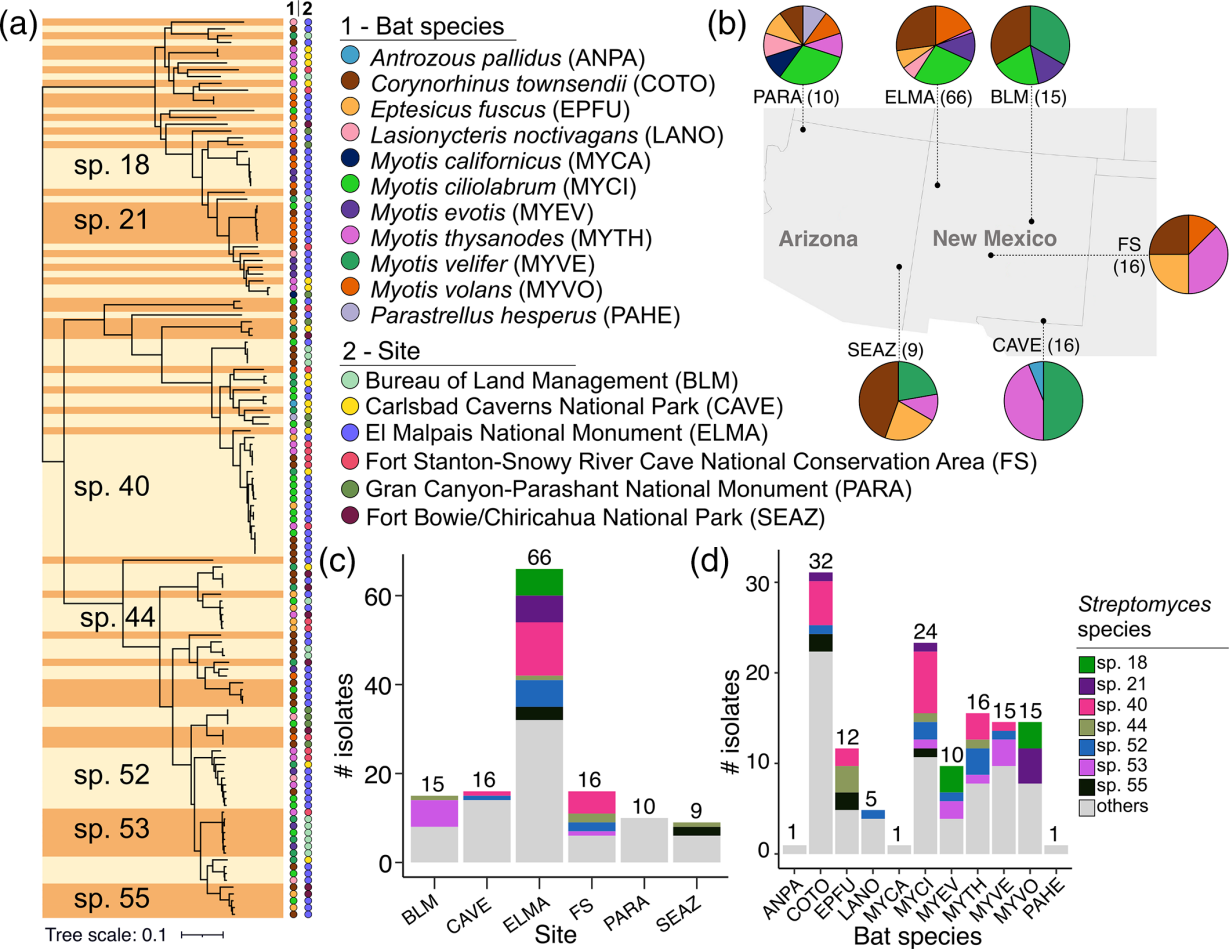
Species diversity and geographical distribution of bat-associated *Streptomyces*. (**a**) Maximum likelihood phylogenetic tree built using 297 305 single nucleotide polymorphisms (SNPs) in an alignment of 620 core genes. The tree was rooted at its midpoint. Tree scale represents the number of nucleotide substitutions per site. The alternating orange and yellow strip represent the species boundaries calculated using the genome-wide average nucleotide identity (ANI). For visual clarity, only the species represented by more than five genomes are labelled (sp. 18, 21, 40, 44, 52, 53, 55). Coloured dots next to the tree indicate the bat species (labelled 1) and site (labelled 2) from which the isolate was obtained. (**b**) The six sites from where the bats were sampled. For each site, a pie chart shows the distribution of the different bat species and the number of *Streptomyces* isolates collected is shown in parenthesis. Bar plots showing the number of *Streptomyces* isolates from each site (**c**) and bat species (**d**). The seven most frequently detected species are represented in coloured blocks. The colours of bat species and sites are identical in all four panels and correspond to the colour legend in panel A. For visual clarity, we used acronyms to represent the names of bat species and sites. Bat species and site abbreviations are defined in Fig. 1.

We obtained whole genome sequences of 132 *Streptomyces* isolates sampled from the skin and fur surfaces of healthy bats (Table S1). These consisted of 73 previously published genomes [21] and 59 sequenced in the current study. The isolates came from insectivorous bats representing six genera and 11 species: pallid bat (*Antrozous pallidus*; ANPA), Townsend’s big-eared bat (*Corynorhinus townsendii*; COTO), big brown bat (*Eptesicus fuscus*; EPFU), silver-haired bat (*Lasionycteris noctivagans*; LANO), California bat (*Myotis californicus*; MYCA), western small-footed bat (*Myotis ciliolabrum*; MYCI), western long-eared bat (*Myotis evotis*; MYEV), fringed bat (*Myotis thysanodes*; MYTH), cave bat (*Myotis velifer*; MYVE), long-legged bat (*Myotis volans*; MYVO), and canyon bat (*Parastrellus hesperus*; PAHE) (Fig. 1a). These were collected from two caves sites in Arizona (Grand Canyon-Parashant National Monument [PARA] and Fort Bowie/Chiricahua National Park [SEAZ]) and four sites in New Mexico (El Malpais Conservation Area [ELMA], Bureau of Land Management caves 45 and 55 [BLM], Fort Stanton-Snowy River Cave National Conservation Area [FS], and Carlsbad Caverns National Park [CAVE]) (Fig. 1b). The number of contigs per genome ranged between 27–600 (median=169), N50 contig length values between 24 857–1 491 574 bp (median=108 954 bp), and genome length between 7.2–11.1 Mbp (median=9 Mbp) (Fig. S1A–C and Table S1). The GC content ranged from 69.64–72.92 % (median=70.96 %) (Fig. S1D and Table S1), which is typical of *Streptomyces* genomes [44,45].

Using the genome-wide ANI values of all orthologous genes shared between every pair of genomes [31], we delineated 55 species based on the 95 % ANI threshold (Figs 1a and S2, Table S2). Only seven species, designated as species 18, 21, 40, 44, 52, 53, and 55, were represented by more than five genomes. A total of 32 *Streptomyces* species were represented by a single genome. The core genome phylogenetic tree built from 297305 SNPs in an alignment of 620 core genes revealed an intermingled distribution of the isolates from different bat hosts and cave sites. However, caution must be exercised in making inferences about the phylogenetic distribution of isolates. The numbers of isolates from each bat species and from each site were uneven, but these differences reflect the difficulty in sampling such environments. We note that multiple bat species were present in each of the six sites, ranging from three bat species in CAVE to eight bat species in PARA (Figs 1b and S3).

When considering only the seven *Streptomyces* species with more than five genomes represented in our phylogeny, we observed differences in their geographical distribution (Fig. 1c). For example, sp. 44 was present in BLM, ELMA, FS and SEAZ, sp. 40 and sp. 52 were detected in CAVE, ELMA, and FS, sp. 53 in BLM and FS, and sp. 55 in ELMA and SEAZ. In contrast, sp. 18 and sp. 21 were only found in ELMA. In terms of the bacterial distribution among bat species, all seven *Streptomyces* species were detected in more than one bat species (Fig. 1d). For example, sp. 21 was found in COTO, MYCI, and MYVO. Sp. 40 was detected in COTO, EPFU, MYCI, MYTH, and MYVE. Sp. 52 was obtained from COTO, LANO, MYCI, MYEV, MYTH, and MYVE. Not one *Streptomyces* species was detected in all 11 bat species or all six sites. Overall, these results show a remarkable level of genetic diversity in bat-associated *Streptomyces*.

### The overall genome composition of *Streptomyces* is influenced by the bat host and cave site

**Fig. 2.**
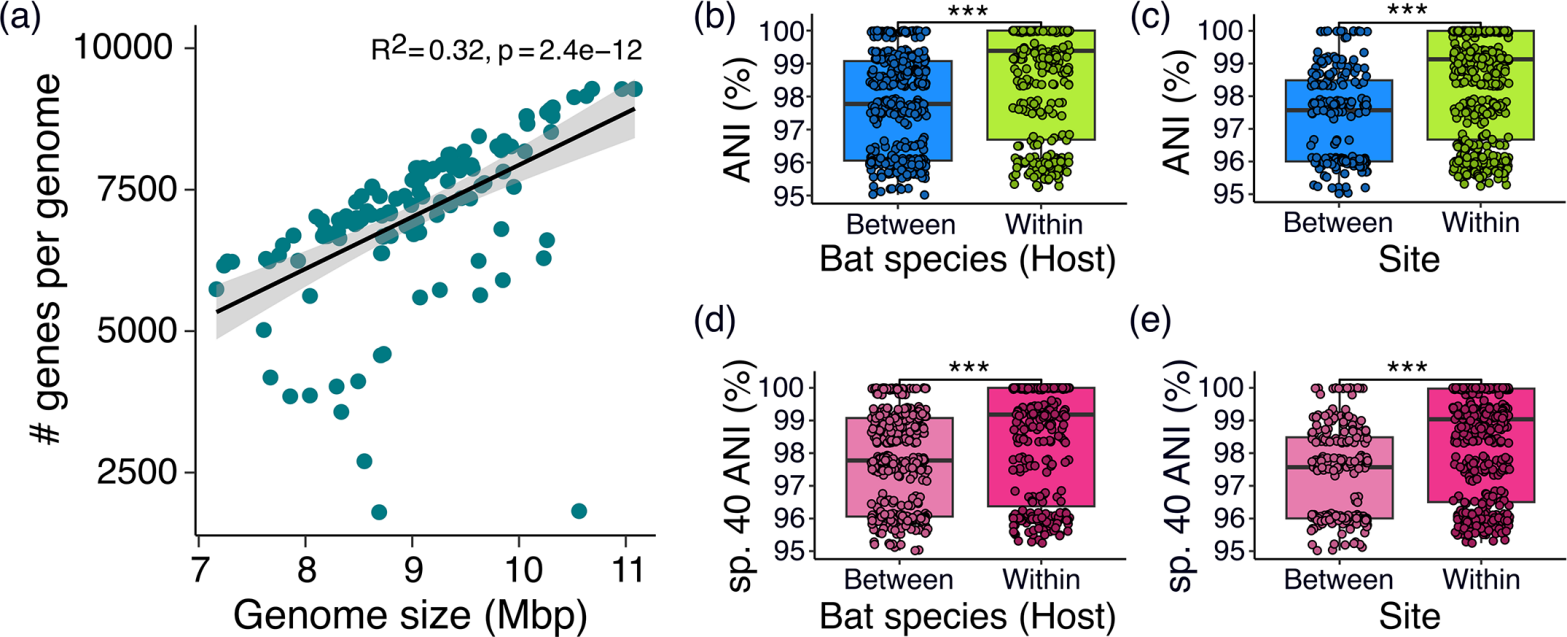
Genomic characteristics of bat-associated *Streptomyces*. (**a**) Correlation between the genome size and the number of genes per genome for isolates randomly chosen from a culture collection of *Streptomyces* from healthy bats (i.e. free of white-nose syndrome) sampled in 2013–2016. Each green dot represents a *Streptomyces* genome. The shaded area surrounding the fitted linear regression line represent the 95 % confidence interval based on the standard error of the mean slope of the regression line. (**b, c**) Pairwise genome-wide average nucleotide identity (ANI) values comparing all pairs of *Streptomyces* isolates sampled (**b**) between the same and different bat species and (**c**) between the same and different sites. (**d, e**) Pairwise genome-wide ANI values comparing all pairs of *Streptomyces* sp. 40 isolates sampled (**d**) between the same and different bat species and (**e**) between the same and different sites. For panels b–e, each dot represents the ANI value for a pair of *Streptomyces* genomes. Box plots show the minimum, first quartile, median, third quartile, and maximum values with outliers depicted as single points. Mann-Whitney U test, *** <0.0001.

The set of protein-coding genes in the entire dataset (or pan-genome [34]) consisted of 620 core genes (i.e. genes present in ≥95 % of genomes), 119119 shared accessory genes (i.e. present between two and 95 % of the genomes), and 43474 singletons (genes present in only one genome) (Table S3). We estimated a mean of 7050 genes per genome, with 6439 accessory genes per genome (including singletons). The number of genes per genome ranged from 1799 to 9284 protein-coding genes per genome (Table S1). We detected a significant positive correlation between the genome size and number of protein-coding genes in *Streptomyces* (R^2^=0.32, *p*-value=2.4e-12; Fig. 2a).

Next, we partitioned the genome-wide ANI values within and between the sources where *Streptomyces* were obtained. Pairwise ANI values were significantly higher between *Streptomyces* genomes from the same bat species compared to those from different bat species (*p*-value=2.2e-16, Wilcoxon rank sum test; Fig. 2b). Pairs of *Streptomyces* from the same bat species have a median ANI of 99.38 %, while pairs of *Streptomyces* obtained from different bat species have a median ANI of 97.78 %. In terms of cave sites, pairwise ANI values were significantly higher between genomes from the same site compared to those from different sites (*p*-value=2.2e-16, Wilcoxon rank sum test; Fig. 2c). Pairs of *Streptomyces* genomes from the same site have a median ANI of 99.13 %, while pairs of *Streptomyces* obtained from different sites have a median ANI of 97.57 %. We also observed similar results when we considered ANI between pairs of genomes of a single *Streptomyces* species. Here, we focus on *Streptomyces* sp. 40, which has the highest number of representative genomes in our dataset (*n*=18 genomes). Pairwise ANI values were significantly higher between *Streptomyces* sp. 40 genomes from the same bat species compared to those from different bat species (*p*-value=1.278e-13, Wilcoxon rank sum test; Fig. 2d) and genomes from the same site than between sites (*p*-value=5.329e-15, Wilcoxon rank sum test; Fig. 2e). These results show that shared ecological source is a major contributor to shaping the overall genome composition of *Streptomyces. Streptomyces* isolates that colonize the same bat species or inhabit the same cave site exhibit greater genomic resemblance than they do with *Streptomyces* from other bat species or sites.

### BGCs are diverse and abundant, but not structured by the source of *Streptomyces*

**Fig. 3.**
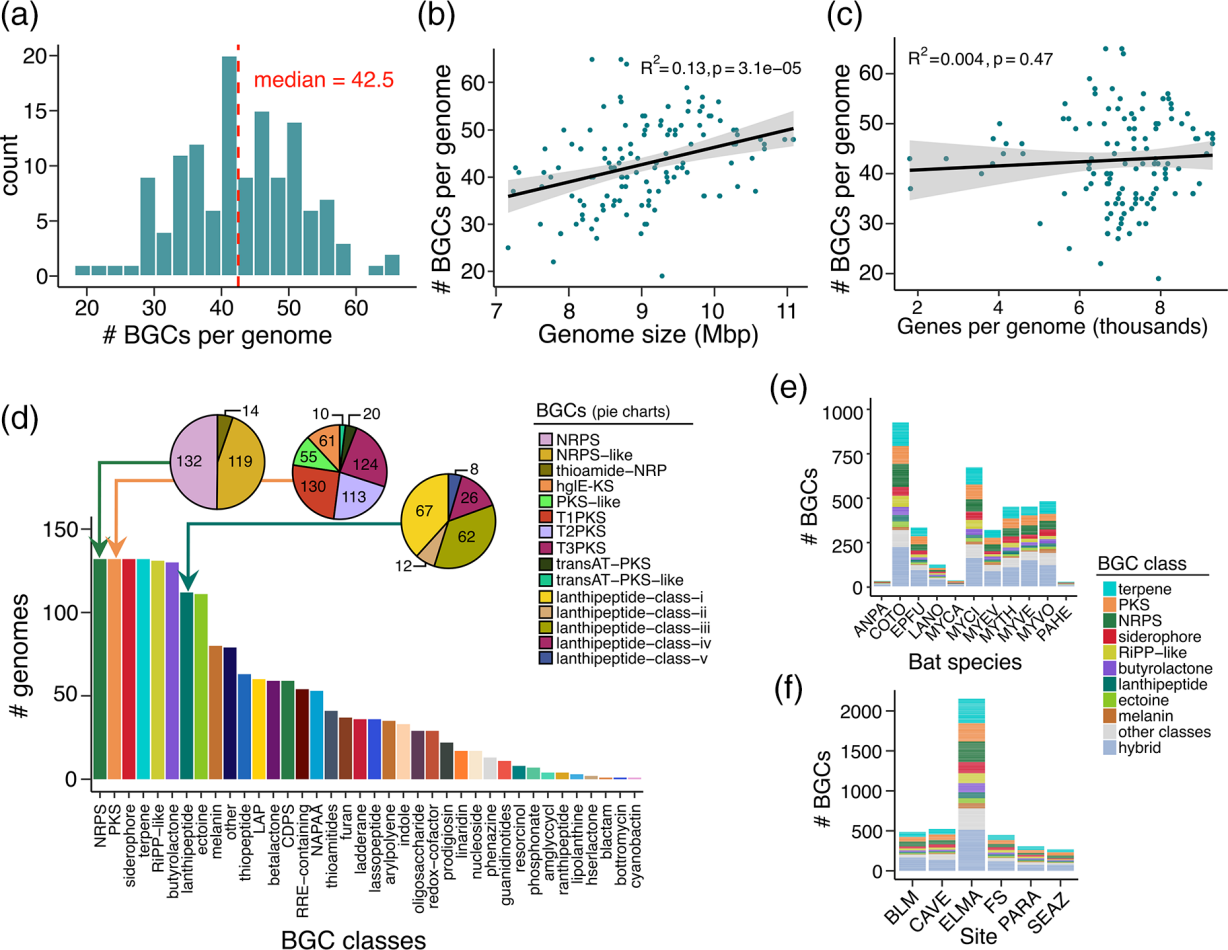
Biosynthetic gene clusters (BGCs) in bat-associated *Streptomyces*. (**a**) Histogram showing the number of BGCs per genome. (**b**) Relationship between the genome size and the number of BGCs per genome. (**c**) Relationship between the number of genes per genome and the number of BGCs per genome. For both panels (b) and (c), each green dot represents a *Streptomyces* genome. The shaded area surrounding the fitted linear regression line represent the 95 % confidence interval based on the standard error of the mean slope of the regression line. (**d**) The number of genomes with at least one BGC from each major class of BGCs. For visual clarity, only the top 38 most common BGC classes are shown. Complete information about the BGCs found in each genome are found in Fig. S4 and Table S4. The number of genomes that carry at least one variant of NRPS, PKS and lanthipeptide are shown in the pie charts. The number of *Streptomyces* BGCs found in each bat species (**e**) and site (**f**). For panels (e) and (f), only the nine most common BGCs are shown. Colours of BGCs in panels (e) and (f) correspond to the colours of bars in panel (d). Bat species and site abbreviations are defined in Fig. 1.

Across our entire dataset, we detected a total of 5647 putative BGCs (Table S4). The number of BGCs per genome greatly varied, even among members of the same species (Fig. S4). The number of BGCs per genome in our dataset ranged from 19 to 65 (median=42.5) (Fig. 3a), which is consistent with those reported in other studies of *Streptomyces* [45,47]. We detected a significant positive correlation albeit small between the genome size and the number of BGCs per genome (R^2^=0.13, *p*-value=3.1e-05; Fig. 3b), but not between the number of protein-coding genes per genome and the number of BGCs per genome (R^2^=0.004, *p*-value=0.47; Fig. 3c).

The BGCs in bat-associated *Streptomyces* can be classified into 50 major classes (Fig. S4 and Table S4). The most common BGC classes were the non-ribosomal peptide synthetase (NRPS), polyketide synthase (PKS), siderophore, and terpene, all of which were detected in all genomes at least once (Fig. 3d). BGCs found in fewer than ten genomes included resorcinol (eight genomes), phosphonate (seven genomes), aminoglycoside/aminocyclitol (four genomes), ranthipeptide (four genomes), lipolanthine (three genomes), homoserine lactone (two genomes), beta-lactam (one genome), bottromycin (one genome), and cyanobactin (one genome). The number of copies of a BGC from a specific class varied between genomes. We found a median of four terpenes (range: 2–11 copies) per genome, three NRPS (range: 0–9 copies) per genome, three PKS (range: 0–10 copies) per genome, two butyrolactones (range: 2–4) per genome, two ribosomally synthesized and post-translationally modified peptide products (RiPP-like; range: 0–4) per genome, and two siderophores (range: 1–5) per genome.

Some of the BGCs can be further classified into structural variants (Fig. 3d). For example, NRPS can be distinguished into NRPS only (present in 132 genomes), NRPS-like (119 genomes), and thioamide-NRPS (14 genomes). Type 1 (T1PKS), Type 2 (T2PKS) and Type 3 (T3PKS) were the most frequent variations of PKS and were detected in 130, 124 and 113 genomes, respectively. Four other variants of PKS were also detected in *Streptomyces* genomes. Of the five classes of lanthipeptides, classes I (lanthipeptides like nisin) and III (lanthipeptides like labyrinthopeptin) were the most frequently detected (67 and 62 genomes, respectively).

When we subdivided the *Streptomyces* BGCs according to bat species, we detected seven BGCs present in ten of the 11 bat species (Fig. 3e). These include terpene, PKS, NRPS, siderophore, RiPP-like, butyrolactone, lanthipeptide, ectoine, and melanin. We did not find a significant difference in the number of *Streptomyces* BGCs across all bat host species (Fig. S5A; *p* = 0.097, ANOVA). When we categorized the *Streptomyces* BGCs according to site, we found BGC classes that were present in all six sites (Fig. 3f). These include terpene, PKS, NRPS, siderophore, RiPP-like, butyrolactone, lanthipeptide, ectoine, and melanin. The number of *Streptomyces* BGCs was significantly different among the six sites (*P*=0.18, ANOVA) (Fig. S5B). We also did not find any significant correlation between the number of accessory genes and the number of BGCs per genome when we subdivided the genomes according to bat species and site (Fig. S6).

These results show that bat-associated *Streptomyces* harbour a highly diverse repertoire of BGCs, with major BGC classes widely distributed across different bat species and cave sites.

### Hybrid BGCs expand the biosynthetic potential of *Streptomyces*

**Fig. 4.**
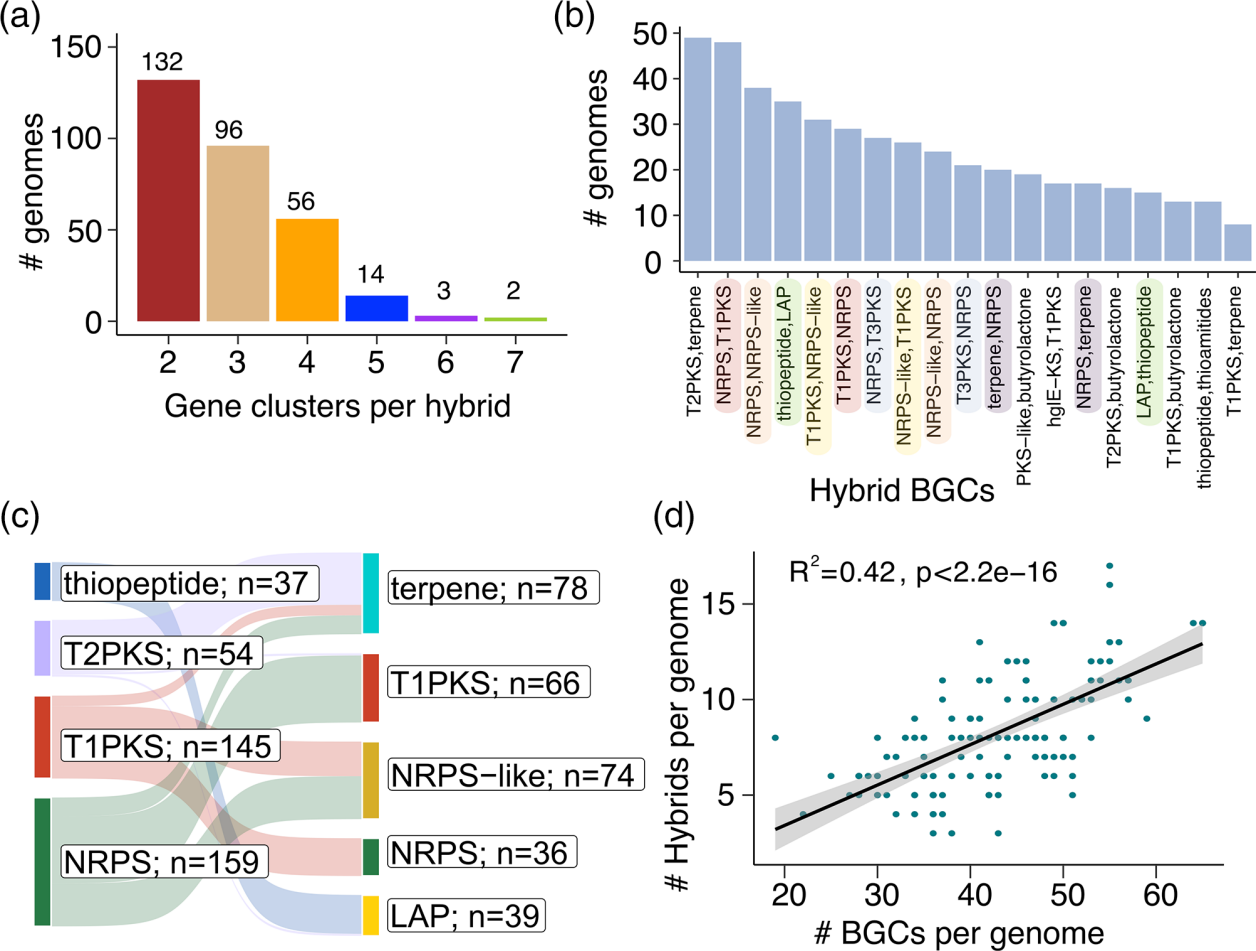
Hybrid biosynthetic gene clusters (BGCs) bat-associated *Streptomyces*. (**a**) Number of *Streptomyces* genomes that carry hybrid BGCs with 2-, 3-, 4-, 5-, 6- and 7-BGC domains. (**b**) Number of genomes carrying the 20 most common hybrid BGCs. Hybrid names with the same colours have the same BGC domain, but differ in the linear order of the domains. (**c**) Sankey plot showing the different combinations of BGC domains in the five most common hybrid BGCs. The first column of colours represents the first BGC domain in a linear order of domains within a hybrid BGC, while the second column of colours represent the second BGC domain. The colours are identical to those found in Fig. 3d. Connecting lines represent the different combinations of BGC domains. The numbers represent the number of hybrid BGCs that contain each BGC domain. (**d**) The relationship between the number of BGCs per genome and the number of hybrids per genome. Each green dot represents a *Streptomyces* genome. The shaded area surrounding the fitted linear regression line represent the 95 % confidence interval based on the standard error of the mean slope of the regression line.

Hybrid BGCs contain genes that code for signature domains of more than one distinct class of BGC [48,50]. The structural and chemical modifications in hybrid BGCs lead to the production of derivatives of a secondary metabolite, thus greatly expanding the chemical repertoire of a bacterium [5,48]. Across the 132 bat-associated *Streptomyces*, we detected a total of 1086 hybrid BGCs, of which 353 were unique combinations of hybrid BGCs (Table S5). Hybrid BGCs were identified in all genomes, regardless of bat species or cave sites (Fig. S4). The median number of hybrid BGCs per genome was eight (range: 3–17). We found hybrid BGCs consisting of signature domains from two to seven distinct BGC classes (Fig. 4a). The most common were hybrids with two domains, which we detected in 132 genomes. The least common hybrids were those that contain six or seven domains. Only two genomes contain seven-domain hybrids (sp. 8 from *Myotis ciliolabrum* in BLM; sp. 31 from *Eptesicus fuscus* in PARA). The most frequently detected two-domain hybrid combinations were T2PKS-terpene (present in 49 genomes) and NRPS-T1PKS (48 genomes) (Fig. 4b). The 3-, 4-, 5-, 6- and 7-domain hybrids were also present but were not as frequently detected as the two-domain hybrids (Fig. S7). A BGC class can form a hybrid with multiple classes of BGCs. For example, NRPS can form a hybrid with terpene, T1PKS, or NRPS-like BGCs. T2PKS can form a hybrid with terpene, T1PKS, and linear azol(in)e-containing peptides (LAP) (Fig. 4c).

Further modifications in hybrid BGCs are derived from alterations in the linear order of each BGC domain in the chromosome, which can alter the order of synthesis of compounds and their structural derivatives [51,52]. For example, we found NRPS-T1PKS and T1PKS-NRPS in 48 and 29 genomes, respectively. Thiopeptide-LAP and LAP-thiopeptide were detected in 35 and 15 genomes, respectively. Lastly, we detected a significant positive correlation between the number of BGCs per genome and the number of hybrid BGCs per genome (R^2^=0.42, *p*-value <2.2e-16; Fig. 4d). The origin and mechanisms involved in hybrid BGC formation remain unclear, but it is notable that the presence of hybrid BGCs greatly expands the biosynthetic repertoire of *Streptomyces*.

## Discussion

A crucial approach to addressing global health threats and sustaining industrial production is to maximize our ability to explore the chemical diversity already found in nature and understand how it arose. *Streptomyces* bacteria are a fertile source of metabolites with immense utility in clinical and non-clinical settings. Here, we characterized the genomic and biosynthetic diversity of 132 *Streptomyces* isolates sampled from 11 species of insectivorous bats across six cave sites in Arizona and New Mexico, USA.

We highlight two major findings. First, bat-associated *Streptomyces* are remarkably diverse. From the 132 isolates sequenced, a total of 55 species can be delineated, of which 32 species are represented by a single genome. Despite the recorded diversity of *Streptomyces* [53,54], the largest phylum in the domain Bacteria, new species continue to be discovered. Our findings expand the current knowledge of the breadth of vertebrate niches of *Streptomyces*. In humans, rare species of *Streptomyces* cause chronic subcutaneous infection [55] and respiratory infection [56]. Our group has pioneered sampling efforts of bacteria from bats across the United States [18,57,59]. Many of the bat-associated *Streptomyces* may therefore likely represent novel species. Future work exploring *Streptomyces* in other underexplored environments may identify additional novel species. For example, it would be interesting to elucidate how different types of caves (e.g. lava cave, karst cave), type of rock, bat species from other geographical regions, and bat behaviours influence the assemblage of *Streptomyces* species and their genomic elements. Reconstructing the evolutionary history of bat-associated *Streptomyces* will bring important insights on the ancient origins, ancestral lifestyles, and the long-term stability of these bacteria in bats, including any signature of co-evolution between them.

Second, cave sites and bat host species influence the genomic diversity but did not affect the distribution of major BCG classes. The distribution of major BGC classes does not appear to be ecologically structured or restricted; rather, they were widespread across ten of the 11 bat species and in all cave sites. In contrast, *Streptomyces* isolates that colonize the same bat species or inhabit the same site exhibit greater overall genomic similarity than they do with *Streptomyces* from other bat species or sites. This suggests that bat-associated *Streptomyces* benefit from a common pool of BGC-encoded metabolites, yet the rest of their genomes experience continued differentiation in response to adaptation to specific environments. In contrast, soil-dwelling *Streptomyces* experience diversification of their BGCs that coincides with lineage divergence [60]. A more comprehensive analysis of animal-associated versus free-dwelling *Streptomyces* from diverse sources will bring critical insights to the evolutionary and ecological factors that shape the divergence of this pharmaceutically important taxon.

The biosynthetic repertoire of bat-associated *Streptomyces* consisted of remarkably diverse classes of BGCs, which are augmented by the numerous BGC structural variants within each class and hybrid BGCs. Chemical analyses and inhibition assays of these compounds against pathogens will shed light on how *Streptomyces* BGCs can be leveraged and/or manipulated to improve bats’ resistance against *P. destructans*. It is possible that the assemblage of certain *Streptomyces* strains can produce a specific potent or specialized cocktail of antifungals, similar to what has been described in beewolf digger wasps [11]. Such unique combination of antimicrobials is due to the interactions between *Streptomyces* strains that can alter the production of different metabolites depending on the identity of neighbouring strains [61,62]. Future work focused on creating synthetic microbial *Streptomyces* communities, i.e. co-culturing specific taxa under well-defined conditions [63], is an excellent approach to identifying antibiotic cocktails. Moreover, the contributions of *Streptomyces* to bat defence against numerous lineages of ectoparasitic insects, most notably bat flies (Diptera) [64], remain to be elucidated. Bat-fly interactions are specialized and are influenced by the environment [65], and we can speculate that the bacterial composition of bat skin may modulate or restructure the bat’s interactions with insects [66].

The result of our BGC analysis is also an important step in understanding the mechanisms that allow *Streptomyces* to survive on bat skin and fur. However, whether bats are actually colonized by *Streptomyces* (i.e. the bacteria are actively growing on the skin) versus simply harbouring spores or non-growing cells due to environmental exposure remains unclear. The siderophore BGCs are particularly intriguing and may shed light on *Streptomyces* survival on bat skin. All the *Streptomyces* genomes in our analyses carry at least one siderophore BGC. Siderophores are small and low-molecular-weight metabolites that function to sequester and chelate Fe^3+^, and which can also influence microbial social interactions and host cellular iron homeostasis [67,68]. It is a common strategy used by microbes to survive in iron-deficient environments, such as the skin. Future investigations on the structural and functional diversity of siderophores in *Streptomyces* (e.g. ferrioxamine, desferrioxamine, foroxymithine; [69,70]) are needed to understand bacterial adaptive strategies in bats. We can postulate that patterns of siderophore production will vary under different environmental conditions, for example, in instances when bats are exposed to pathogens or harsh conditions, different bat activities such as foraging and hibernation, or differences in caves that bats occupy.

We recognize the limitations of the current study. First, our sampling scheme involves only a single *Streptomyces* isolate obtained from an individual bat. As shown previously in other studies, there is considerable microbial diversity within individual bats [66,71]. Such within-host diversity of bacterial strains may contribute to better understanding of disease susceptibility and/or resistance and the overall health of the bat host. Second, *in silico* gene prediction, including BGC identification, is affected by both the composition of sequence databases used for comparison and the quality of query sequences. *Streptomyces* genomes are extremely large, exceptionally GC-rich, and contain many highly repetitive DNA sequences [72,73], all of which pose obstacles in obtaining a high quality genome assembly when using short-read sequencing methods. Contig breakpoints are frequently associated with highly repetitive sequences [74], which are present in *Streptomyces* genomes [72,73] and tend to be associated with secondary metabolism [75]. The unavoidable consequence of this is that the BGC count is usually elevated for draft genomes compared to if they were closed. Although our BGC estimates were consistent with previous reports of *Streptomyces* BGCs [45,47], we recognize that our use of short-read sequencing can influence BGC estimates. Short-read assembly polished with long-read sequencing should considerably improve future *Streptomyces* sequencing efforts.

In conclusion, we show that the importance of bat hosts and geographical sites in shaping the exceptional *Streptomyces* diversity. Overlapping niches in terms of cave sites and bat host species influence the overall genomic diversity of *Streptomyces* bacteria but did not influence the distribution of major BGCs classes. Taken together, our results bring critical insights to understanding *Streptomyces*-bat ecology and BGC diversity that may contribute to bat health and in augmenting current efforts in natural product discovery, especially from underexplored or overlooked environments.

## supplementary material

10.1099/mgen.0.001238 Uncited Fig. S1.

10.1099/mgen.0.001238 Uncited Table S1.

## Abbreviations

ANI: average nucleotide identity
BGC: biosynthetic gene cluster
NRPS: non-ribosomal peptide synthetase
PKS: polyketide synthase
