# Supplementary material for: Ecology shapes the genomic and biosynthetic diversification of Streptomyces bacteria from insectivorous bats
Source: Microb Genom. 2024 Apr 16;10(4):001238. doi: 10.1099/mgen.0.001238 (PMC11092124; doi:10.1099/mgen.0.001238)
Supplement: Uncited Fig. S1. [file mgen-10-01238-s001.pdf]

Ecology shapes the genomic and biosynthetic diversification of *Streptomyces* bacteria from insectivorous bats

Manuela Montoya-Giraldo[1], Odion O. Ikhimiukor[1], Cooper J. Park[2], Nicole A. Caimi[3], Debbie C. Buecher[4], Ernest W. Valdez[3,5], Diana E. Northup[3#], Cheryl P. Andam[1#]

[1] Department of Biological Sciences, University at Albany, State University of New York, Albany, New York, USA
[2] Department of Molecular, Cellular and Biomedical Sciences, University of New Hampshire, Durham, New Hampshire, USA
[3] Department of Biology, University of New Mexico, Albuquerque, New Mexico, USA
[4] Buecher Biological Consulting, Tucson, Arizona, USA
[5] U.S. Geological Survey, Fort Collins Science Center, Fort Collins, Colorado, USA

# Address correspondence to:
Cheryl P. Andam: candam@albany.edu
Diana E. Northup: dnorthup@unm.edu

Any use of trade, firm, or product names is for descriptive purposes only and does not imply endorsement by the U.S. Government.

SUPPLEMENTARY FILES

Supplementary Table S1. Accession numbers, metadata and genome characteristics of 132 *Streptomyces* genomes for isolates randomly chosen from a culture collection of *Streptomyces* from healthy bats (*i.e.*, free of white-nose syndrome) sampled in 2013–2016. CDS – coding sequence.

Supplementary Table S2. Average nucleotide identity (ANI) values for all pairs of genomes of *Streptomyces* isolates calculated using fastANI. Isolates were randomly chosen from a culture collection of *Streptomyces* from healthy bats (*i.e.*, free of white-nose syndrome) sampled in 2013–2016.

Supplementary Table S3. Pan-genome analysis of the 132 *Streptomyces* genomes estimated using Panaroo. Isolates were randomly chosen from a culture collection of *Streptomyces* from healthy bats (*i.e.*, free of white-nose syndrome) sampled in 2013–2016. QC – quality control

Supplementary Table S4. Biosynthetic gene clusters (BGCs) of the 132*Streptomyces* genomes estimated using antiSMASH. Isolates were randomly chosen from a culture collection of *Streptomyces* from healthy bats (*i.e.*, free of white-nose syndrome) sampled in 2013–2016

Supplementary Table S5. Hybrid biosynthetic gene clusters (BGCs) identified in *Streptomyces* genomes for isolates randomly chosen from a culture collection of *Streptomyces* from healthy bats (*i.e.*, free of white-nose syndrome) sampled in 2013–2016. For visual clarity, each hybrid is split into its component BGC domains.

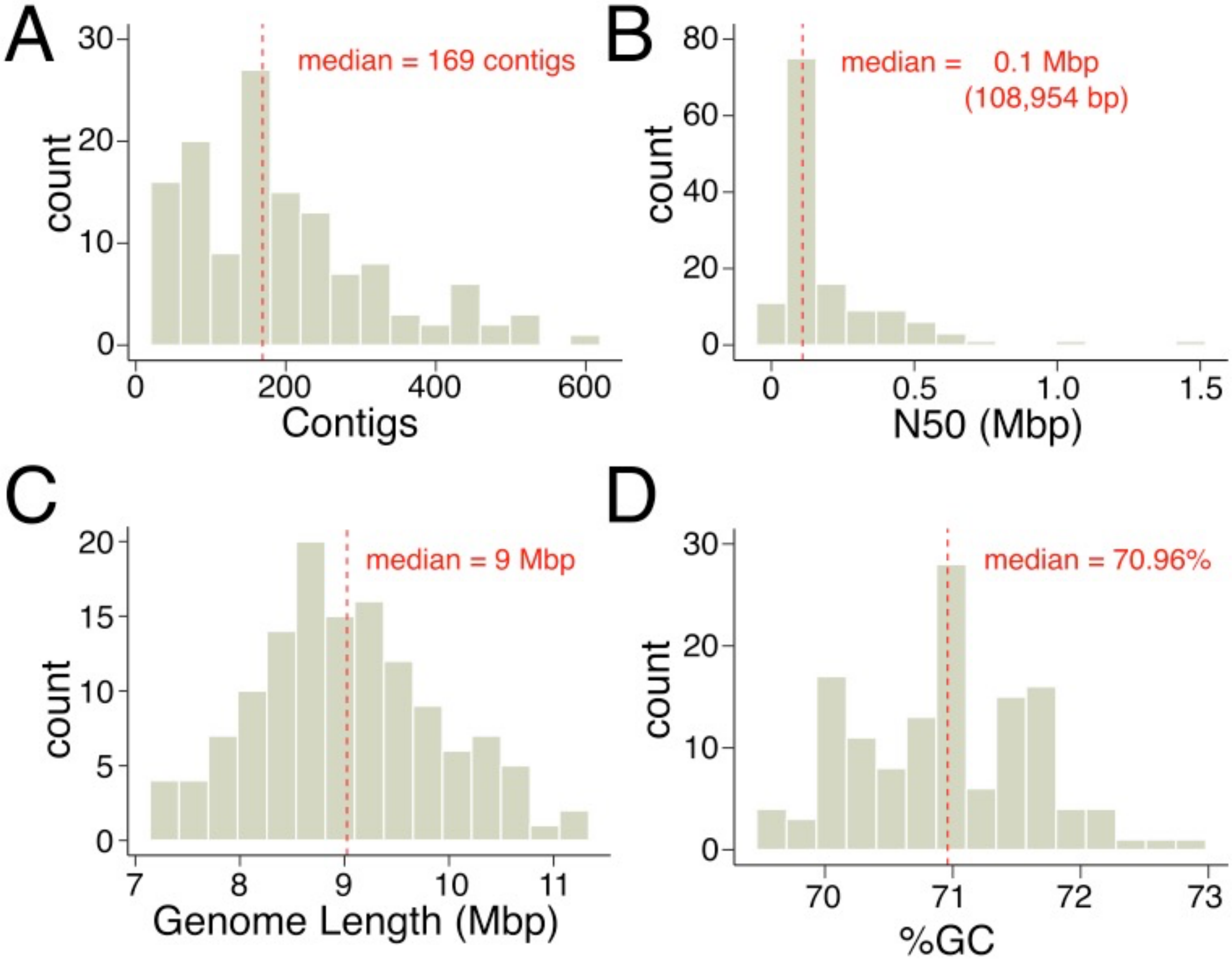

Supplementary Figure S1. Genome characteristics of the 132 *Streptomyces* isolates randomly chosen from a culture collection of *Streptomyces* from healthy bats (*i.e.*, free of white-nose syndrome) sampled in 2013–2016. A. Density distribution of (A) number of contigs; (B) N50; (C) Genome length; and (D) GC content.

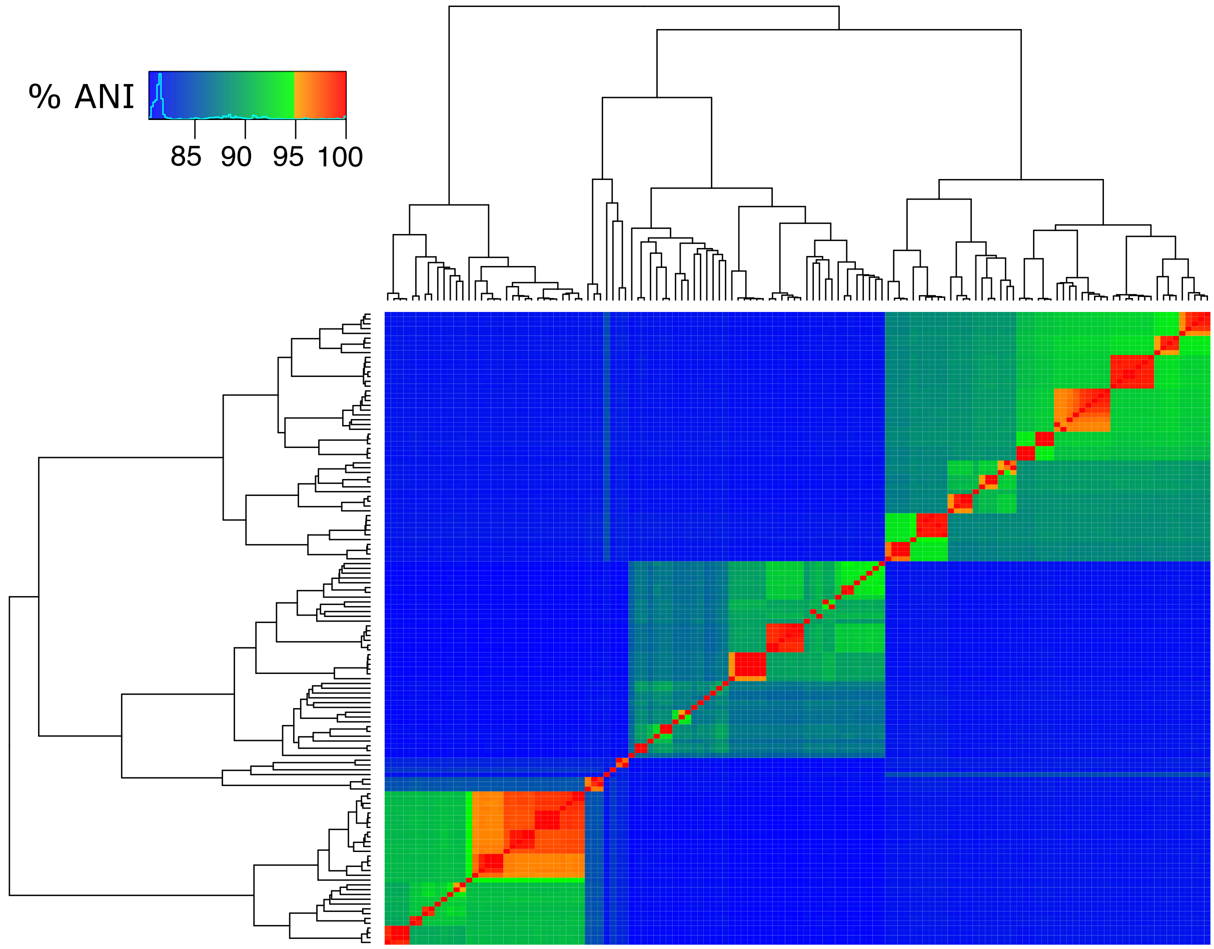

Supplementary Figure S2. Pairwise matrix of Average nucleotide identity (ANI) values calculated using fastANI for isolates from a culture collection of *Streptomyces* from healthy bats (*i.e.*, free of white-nose syndrome) sampled in 2013–2016. Two genomes are considered members of the same species if their ANI is >= 95%.

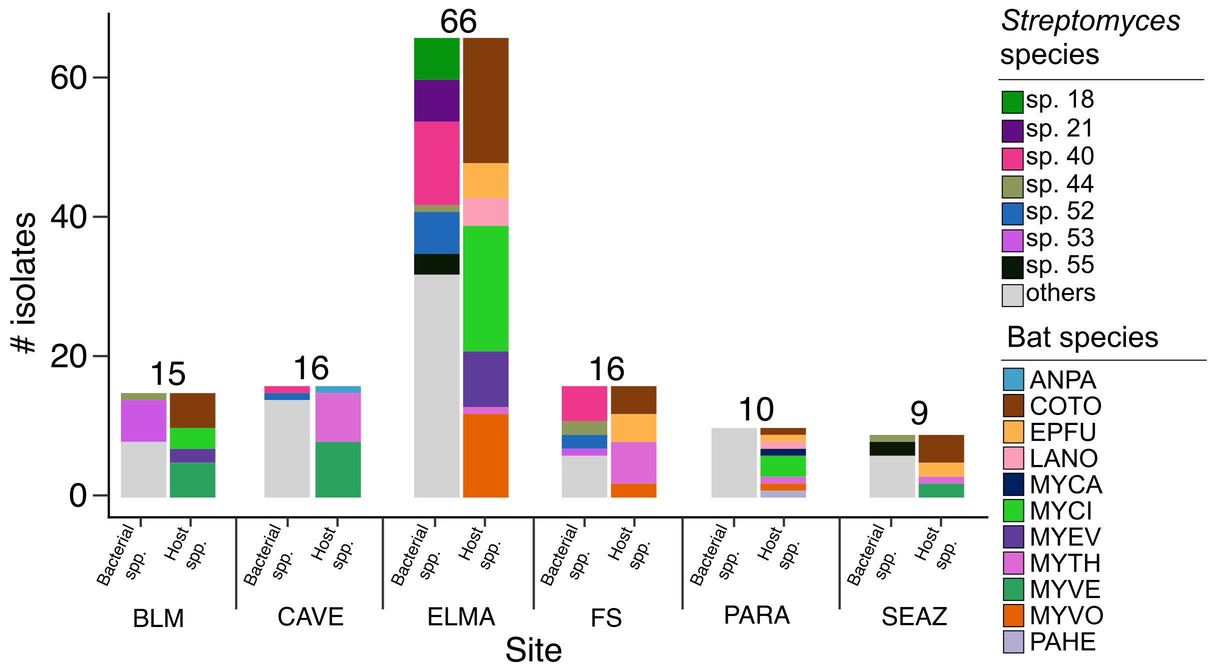

Supplementary Figure S3. Distribution of the seven *Streptomyces* species (*i.e.*, species represented by more than five genomes; Figure 1A) for isolates from a culture collection of *Streptomyces* from healthy bats (*i.e.*, free of white-nose syndrome) sampled in 2013–2016 and bat species across six sites. Bat species and site abbreviations are defined in Figure S4.

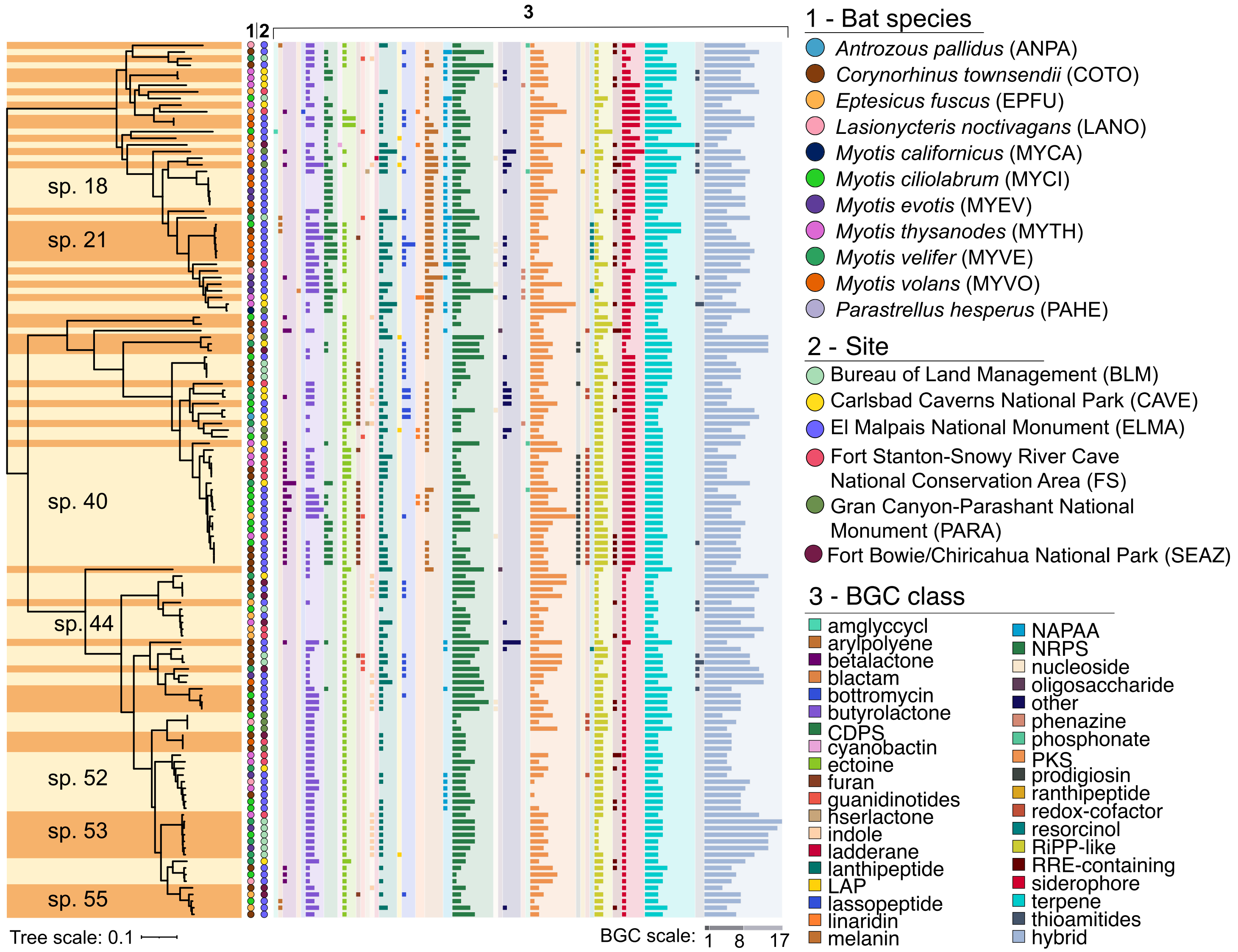

Supplementary Figure S4. Phylogenetic distribution and abundance of biosynthetic gene clusters (BGCs) among the 132 *Streptomyces* genomes. Colors of BGC classes are identical to those in Figure 3D-F. The core genome phylogenetic tree is identical to that in Figure 1A. Midpoint-rooting was used to root the tree. Scale bar represents the number of nucleotide substitutions per site.

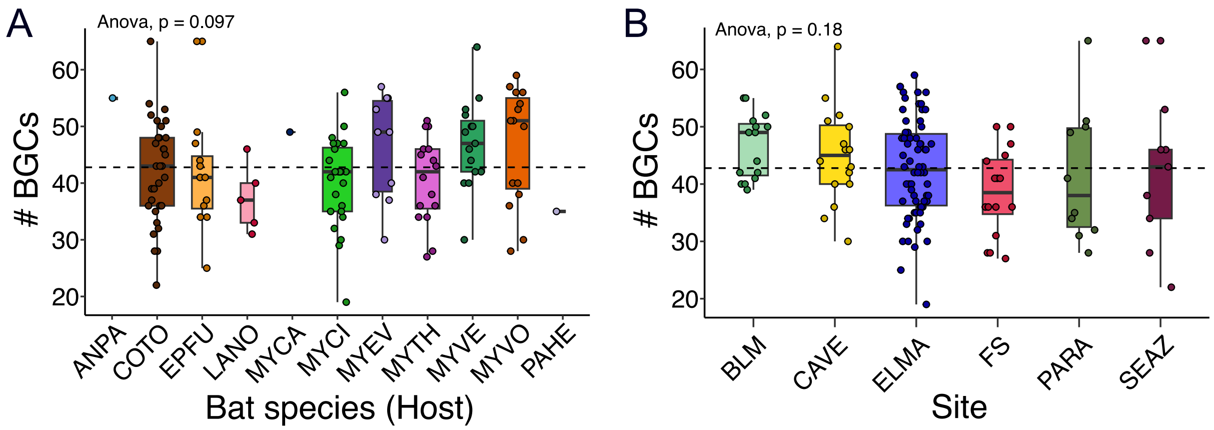

Supplementary Figure S5. Comparison of the number of biosynthetic gene clusters (BGCs) per genome classified according to (A) bat species and (B) site. Each dot represents a *Streptomyces* genome. Box plots show the minimum, first quartile, median, third quartile, and maximum values with outliers depicted as single points. The dotted line represents the overall mean used for multiple comparisons. A global statistical test was calculated using ANOVA and the p-values for ANOVA are shown on the top right of each plot. A pairwise comparison between subsets of Streptomyces was also carried out using Mann-Whitney U test (or Wilcoxon rank sum test) and the significance for this test is represented as asterisks. For visual clarity, only the significant results for the Mann-Whitney U test are shown. Only the *Streptomyces* from the Bureau of Land Management (BLM) and El Malpais Conservation Area (ELMA) are significantly different from each other (p-value = 0.0017). Abbreviations are defined in Figure S4.

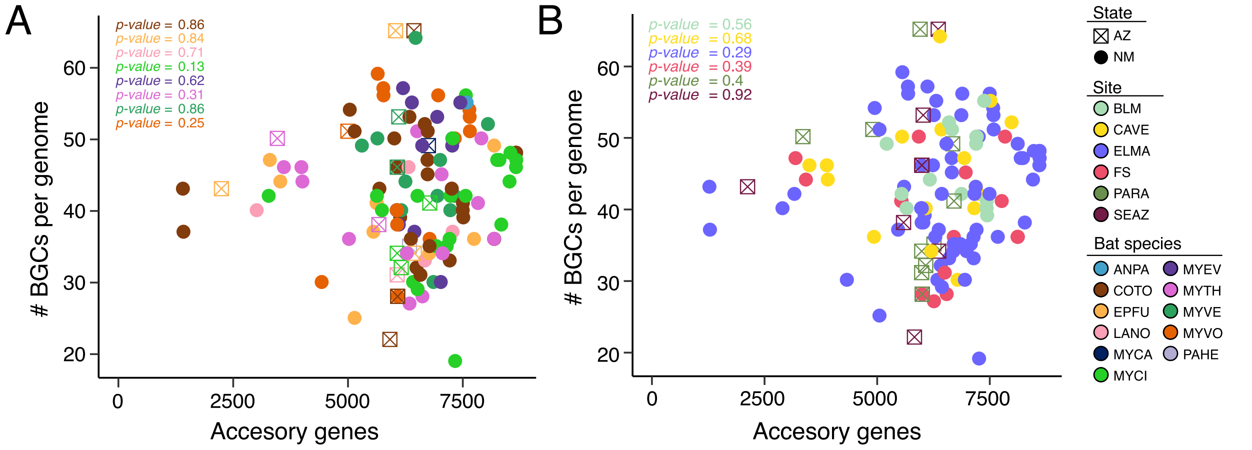

Supplementary Figure S6. Relationship between the number of accessory genes per genome and the number of biosynthetic gene clusters (BGCs) per genome. Both plots are identical but are colored according to (A) bat species and (B) site. The coefficient of determination (R^2) was calculated for each *Streptomyces* subset according to their ecological source and the p-values are colored accordingly. Abbreviations are defined in Figure S4.

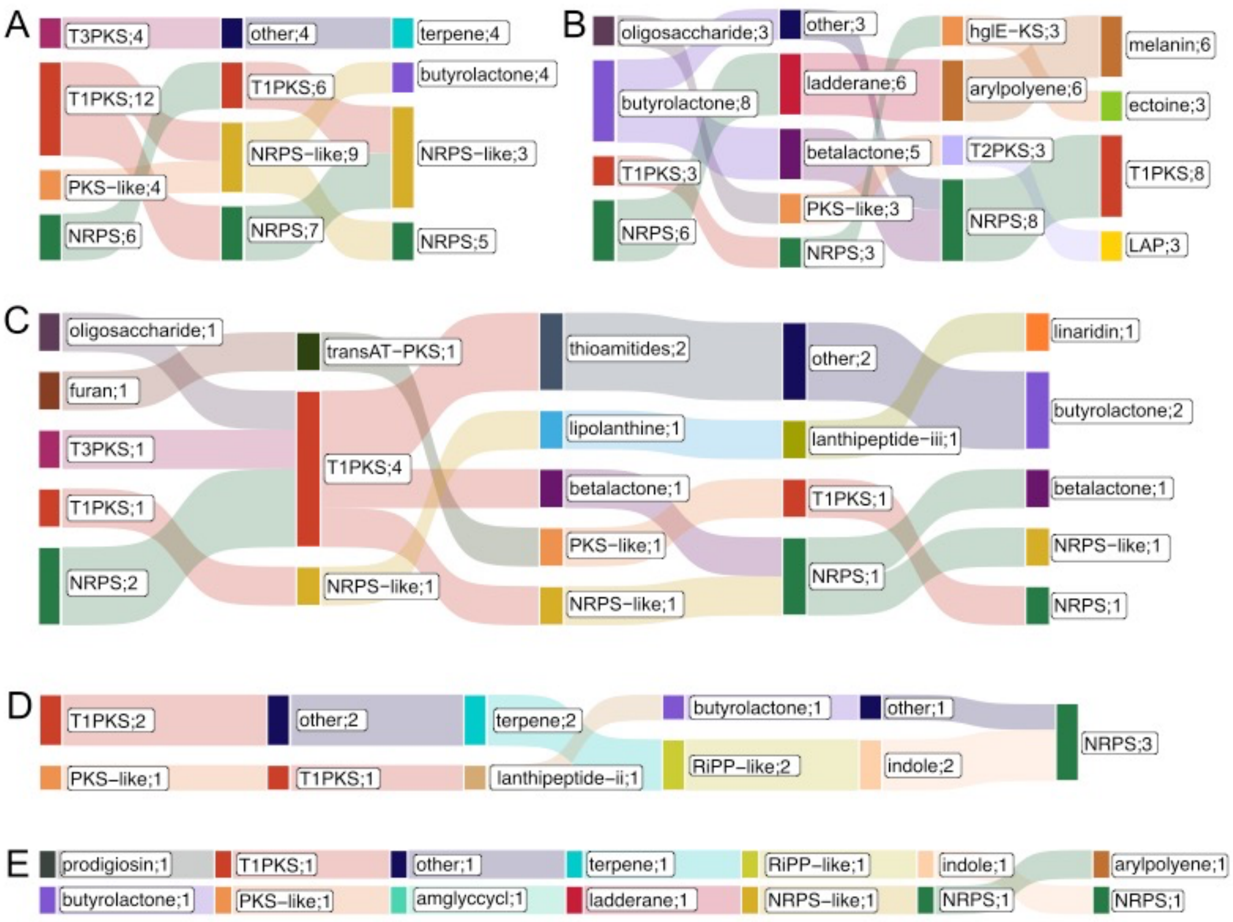

Supplementary Figure S7. Sankey plots showing the most common hybrid biosynthetic gene clusters (BGCs) containing (A) three domains, (B) four domains, (C) five domains, (D) six domains, and (E) seven domains. The order of the BGC domains in each plot reflects the position of the individual domain in a hybrid BGC and the connecting lines indicate the sequential position of each BGC domain. Colors of BGC classes are identical to those in Figure 3D. The numbers next to each BGC name represent the numbers of hybrids that contain a specific BGC.
